# BET protein inhibition in macrophages enhances dorsal root ganglion neurite outgrowth in female mice

**DOI:** 10.1002/jnr.25036

**Published:** 2022-02-26

**Authors:** Georgina Palomés‐Borrajo, Xavier Navarro, Clara Penas

**Affiliations:** ^1^ Department of Cell Biology, Physiology and Immunology, Centro de Investigación Biomédica en Red sobre Enfermedades Neurodegenerativas (CIBERNED) Institute of Neurosciences, Universitat Autònoma de Barcelona Bellaterra Spain

**Keywords:** BET proteins, cytokines, inflammation, neurite outgrowth, regeneration

## Abstract

Peripheral nerve regeneration is limited after injury, especially in humans, due to the large distance the axons have to grow in the limbs. This process is highly dependent on the expression of neuroinflammatory factors produced by macrophages and glial cells. Given the importance of the epigenetic BET proteins on inflammation, we aimed to ascertain if BET inhibition may have an effect on axonal outgrowth. For this purpose, we treated female mice with JQ1 or vehicle after sciatic nerve crush injury and analyzed target reinnervation. We also used dorsal root ganglion (DRG) culture explants to analyze the effects of direct BET inhibition or treatment with conditioned medium from BET‐inhibited macrophages. We observed that although JQ1 produced an enhancement of IL‐4, IL‐13, and GAP43 expression, it did not have an effect on sensory or motor reinnervation after crush injury in vivo. In contrast, JQ1 reduced neurite growth when interacting directly with DRG neurons ex vivo, whereas conditioned medium from JQ1‐treated macrophages promoted neurite outgrowth. Therefore, BET‐inhibited macrophages secrete pro‐regenerative factors that induce neurite outgrowth, and that may counteract the direct inhibition of BET proteins in neurons in vivo. Finally, we observed an activation of the STAT6 pathway in DRG explants treated with conditioned medium from JQ1‐treated macrophages. In conclusion, this study demonstrates that BET protein inhibition in macrophages provides a mechanism to enhance axonal outgrowth. However, specific targeting of BET proteins to macrophages will be needed to efficiently enhance functional recovery after nerve injury.

## Graphical abstract

Treatment with the BET inhibitor JQ1 does not enhance sensory and motor regeneration after sciatic nerve crush injury in female mice (Top). Direct neuronal BET inhibition reduces neurite outgrowth in DRG explants (Middle). However, BET‐inhibited macrophages secrete pro‐regenerative factors, probably anti‐inflammatory cytokines that enhance STAT 6 phosphorylation and neuritogenesis ex vivo (Bottom).

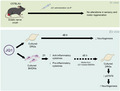

**Significance**

We have found that inhibition of BET proteins in macrophages produce the expression of pro‐regenerative factors by these cells. These factors promote neuronal neurite growth. This finding may be important to promote nerve regeneration after peripheral nerve injury.

## 1 INTRODUCTION

Peripheral nerve injuries (PNIs) result in the disconnection between the neuronal soma and the distal axon stump, leading to the degeneration of distal fibers and the eventual death of axotomized neurons. As a result, there is a partial or total loss of sensory, motor, and autonomic functions (Allodi et al., 2012; Navarro et al., 2007). PNI affects 1.8% of subjects with traumatic limb injuries in Europe. Patients undergo prolonged rehabilitation and current treatments produce a scarce functional outcome (Huckhagel et al., 2018). Hence, it is important to find novel strategies that promote regeneration and recovery after PNI. In fact, peripheral nerve regeneration is limited after injury, especially in humans, due to the large distances the axons have to grow. One way to overcome this problem is by promoting an acceleration of axonal regeneration (DeFrancesco‐Lisowitz et al., 2015; Ma et al., 2011).

During Wallerian degeneration of the distal stump, a network of different cells, including infiltrating macrophages and Schwann cells, participate in axonal and myelin breakdown, through the release of different products. Between these molecules, cytokines and chemokines are essential to regulate the microenvironment that promotes axonal regeneration (Chen, Lin, et al., 2016; DeFrancesco‐Lisowitz et al., 2015). Thus, Wallerian degeneration is a cascade of events that reassemble an immune‐like reaction, which can be divided in two main phases. The early phase is characterized by the synthesis of inflammatory cytokines and chemokines such as TNF‐α, IL‐1α, IL‐1ß, IL‐6, and CCL2, inducing the recruitment of immune cells. The late phase is defined by the increase in anti‐inflammatory cytokines such as IL‐13, IL‐10, and IL‐4, and promotes the clearance of debris (Rotshenker, 2011; Vidal et al., 2013). Both processes are indispensable for successful nerve regeneration. For instance, it has been demonstrated that the enhancement of the anti‐inflammatory phase attenuates pro‐inflammatory cytokine secretion, concludes degeneration, and increases axonal outgrowth (Fregnan et al., 2012; Siqueira Mietto et al., 2015; Vidal et al., 2013). Thus, to provide a favorable environment for axonal growth, it is crucial to achieve a rapid transition from the pro‐inflammatory phase toward the anti‐inflammatory phase of Wallerian degeneration. Experimental evidence has shown that cytokines trigger JAK/STAT signaling pathways, which are associated with neural plasticity. Particularly, IL‐4 and IL‐13 activate STAT6, leading to neuroprotection (Bhattarai et al., 2016; Deboy et al., 2006; Vidal et al., 2013). IL‐6 and IL‐10 activate the STAT3‐mediated pathway, increasing regeneration and neuroprotection of cortical neurons (Chen, Lin, et al., 2016; Dubovy et al., 2019).

BET proteins are epigenetic readers of acetylated lysine residues in histone and nonhistone proteins, and are associated with cellular growth, evasion of apoptosis, and inflammatory response (Filippakopoulos & Knapp, 2014). BET family comprises BRD2, BRD3, BRD4, and the testis‐specific protein BRDT. The most studied member of this family is BRD4. BRD4 interacts with an acetylated p65/RELA subunit, triggering the recruitment of p‐TEFb complex and stimulating the transcription of NF‐kß genes (Hajmirza et al., 2018). Inhibition of BET proteins through the small molecule JQ1 reduces the expression of inflammatory cytokines and prevents TNF‐inducible gene expression in macrophages in vitro (Nicodeme et al., 2010). In addition, we have previously found that treatment with the BET inhibitor JQ1 reduces inflammation, increases the presence of anti‐inflammatory cytokines, and enhances functional outcome after spinal cord injury (Sanchez‐Ventura et al., 2019). Given the importance of BET proteins on inflammatory gene expression, we hypothesized that BET inhibition could favor an early conclusion of Wallerian degeneration through the downregulation of pro‐inflammatory cytokines and the upregulation of anti‐inflammatory cytokines. Such a process may enhance axonal growth after PNI via JAK/STAT pathways.

We used the BET inhibitor JQ1 and analyzed its effects after PNI in mice. To further analyze the effects of BET proteins on neuronal outgrowth ex vivo, we treated dorsal root ganglion (DRG) neuronal explants with JQ1. In addition, we used conditioned medium from JQ1‐treated macrophages, and assessed its effect on DRG neurite growth. In summary, this study aims to determine the role of BET protein inhibition on axonal regeneration and functional recovery after PNI.

## 2 MATERIALS AND METHODS

### 2.1 Animals and surgery

All animal procedures were approved by the Universitat Autònoma de Barcelona Animal Experimentation Ethical Committee (code 10306) and followed the European Commission Directive 2010/63/EU on the protection of animals used for scientific purposes. Female C57BL/6J mice (Charles River Laboratories) were used in all the experiments, which were kept under a 12‐hr light cycle and had access to water and food *at libitum*.

To perform nerve injury, 7‐week‐old female mice were anesthetized by intraperitoneal injection of ketamine (90 mg/kg) and xylazine (10 mg/kg) in saline. Sciatic nerve crush injury was performed on the right hind limb at 45 mm distance from the tip of the fourth digit. The lesion consisted on applying a tight pressure sing forceps (Dumont #5) at the sciatic nerve 3 times during 30 seconds, with different orientations each. Then, the muscle and skin were closed in layers. To prevent reopening and later infection of the wound, the skin was secured with staples and iodine was topically applied. Mice were kept in a warm environment until they recovered from anesthesia. Operated animals were intraperitoneally administered with vehicle (saline with 5% DMSO and 5% Tween‐80), or with JQ1 (30 mg/kg, diluted in vehicle) starting at 2 hr (day 0) or at 4 days postoperation (dpo). Each cage of mice contained animals treated with the different conditions, to minimize experimental bias. A total of 27 animals for in vivo studies (15 animals for functional testing and 12 for western blot and qPCR studies) were used.

### 2.2 Functional testing

Evaluation of axonal regeneration and target reinnervation was conducted through noninvasive electrophysiological tests at 14, 21, and 28 dpo (*n* = 5 animals per condition). Prior to the functional test, mice were anesthetized by intraperitoneal injection with 0.04 ml of ketamine/xylazine mixture. For assessment of peripheral nerve conduction, the sciatic nerve was stimulated with single electrical pulses of 0.02‐ms duration up to supramaximal intensity, delivered by needles inserted percutaneously at the sciatic notch. Compound muscle action potentials (CMAPs) of the tibialis anterior and plantar muscles were recorded by means of needle electrodes, amplified and displayed on the oscilloscope to measure the amplitude and the latency of the M wave (Navarro, 2016). For sensory nerve conduction, the digital nerve was stimulated similarly, with short‐duration electrical pulses of increasing intensity delivered at the sciatic notch. The evoked compound nerve action potentials (CNAPs) were recorded distally at the fourth digit. Functional tests were also performed in the contralateral hind limb as control values for each group.

### 2.3 Analysis of skin reinnervation

Animals were intraperitoneally anesthetized with 0.2 ml of 1:1 pentobarbital‐saline mixture at 28 dpo and perfused with 4% paraformaldehyde (PFA) (*n* = 5 animals per condition). The distal plantar pads of the hind paw were carefully excised and postfixed with 4% paraformaldehyde for an hour to be later cryopreserved in sucrose 30% solution. Pads 1, referred as the most distal and medial pads that belong to the innervation territory of the sciatic and saphenous nerves, and Pads 2, referred as the most distal and lateral pads that belong only to the innervation territory of the sciatic nerve, were cut longitudinally at 40 μm thickness in a Leica CM190 cryostat (https://drp8p5tqcb2p5.cloudfront.net/fileadmin/downloads_lbs/Leica%20CM1950/User%20Manuals/Leica_CM1950_IFU_2v1N_en.pdf, RRID:SCR_018061). Free‐floating samples were washed with PBS and PBS‐0.3% Tween, and later incubated overnight at 4°C with PBS‐0.3%Triton, 1.5% normal donkey serum, and rabbit anti‐PGP9.5 antibody (Table 1). Samples were washed with PBS‐0.3% Tween and again incubated overnight at 4°C with PBS 0.3% Triton, normal donkey serum 5% and Alexa fluor 594 donkey anti‐rabbit (Table 1) Pad slices were washed with PBS‐0.3% Tween, PBS, incubated 1/5,000 in DAPI‐PB for 2 min and washed in PB 1x. Samples were then heated at 37°C until dry and mounted with Fluoromount.

**TABLE 1:** Antibody list

Name of primary antibody	Immunogen	Manufacturer, catalog number, RRID, host	Used concentration, application
RT‐97	H‐Neurofilament	DSHB Cat# rt97, RRID:AB_528399, mose, monoclonal	1/200, IHC
PGP 9.5 (UCHL1)	PGP 9.5	(CEDARLANE Cat# CL7756AP, RRID:AB_2792979, rabbit, polyclonal	1/500, IHC
Stat3 (79D7)	Stat3	Cell Signaling Technology Cat# 4904, RRID:AB_331269, rabbit, monoclonal	1/1,000, WB
Phospho‐Stat3 (Tyr705) (D3A7)	Phospho‐Stat3 (Tyr705)	(Cell Signaling Technology Cat# 9145, RRID:AB_2491009), rabbit, monoclonal	1/1,000, WB
Stat6 (D3H4)	Stat6	(Cell Signaling Technology Cat# 5397, RRID:AB_11220421), rabbit, monoclonal	1/1,000, WB
Phospho‐STAT6 (Tyr641) (46H1L12)	Phospho‐STAT6 (Tyr641)	(Thermo Fisher Scientific Cat# 700247, RRID:AB_2532305), rabbit, monoclonal	1/250, WB
Monoclonal Anti‐α‐tubulin antibody produced in mouse	α‐Tubulin	(Sigma‐Aldrich Cat# T9026,RRID:AB_477593) mouse, monoclonal	1/10,000 WB

Name of secondary antibody	Immunogen	Manufacturer, catalog number, RRID, Host	Used concentration, application
Donkey Anti‐Rabbit IgG (H + L) Antibody, Alexa Fluor 594 Conjugated	Rabbit IgG (H + L)	(Molecular Probes Cat# A‐21207, RRID:AB_141637), donkey	1/200, IHC
Donkey Anti‐Mouse IgG (H + L) Antibody, Alexa Fluor 594 Conjugated	Mouse IgG (H+L)	(Molecular Probes Cat# A‐21203, RRID:AB_141633), donkey	1/200, IHC
Goat Anti‐Rabbit IgG (H L)‐HRP Conjugate antibody	Goat Rabbit IgG (H + L)	(Bio‐Rad Cat# 170–6515, RRID:AB_11125142), goat	1/3,000, WB
Goat Anti‐Mouse IgG (H L)‐HRP Conjugate antibody	Goat Mouse IgG (H + L)	(Bio‐Rad Cat# 170–6516, RRID:AB_11125547), goat	1/3,000, WB
DAPI		(Sigma‐Aldrich Cat#D9564‐10MG)	1/5,000 in Pads; 1/1,000 in DRGs

Intraepidermal nerve fiber (IENF) density and the number of Meissner corpuscles were estimated using a Olympus BX51 Fluorescence Microscope (https://www.olympus‐lifescience.com/en/microscope‐resource/primer/techniques/fluorescence/bx51fluorescence/, RRID:SCR_018949). IENFs were counted on the lateral area of the pads, whereas Meissner’s corpuscles are found on the apex. To determine the number of IENFs per mm length, the basal layer was localized by the observation of DAPI staining. The ocular 40× grid was oriented following the basal layer to determine the number of nerve fibers that crossed it. A total of two fields per slice were counted. The number of Meissner's corpuscles was also determined at 400× magnification.

### 2.4 Analysis of mRNA expression in sciatic nerve

Seven days after the crush injury, mice were perfused with sterile saline (*n* = 3 or 4 mice per group). Then, injured sciatic nerves were removed and snap‐frozen. Tissue was homogenized with QIAzol lysis reagent (QIAGEN), and RNA was extracted using the RNeasy Mini Kit (QIAGEN) following the manufacturer's guidelines. RNA was quantified with a Thermo Fisher NanoDrop 1000 Spectrophotometer (http://tools.thermofisher.com/content/sfs/manuals/nd‐1000‐v3.8‐users‐manual‐8%205x11.pdf, RRID:SCR_016517) and reverse‐transcribed using an Applied Biosystems kit (Thermo Fisher Scientific). Then, the expression of target sequences was quantified by RTqPCR using SYBR Green QPCR Master Mix (Agilent Technologies) and the corresponding primers (Table 2). Glyceraldehyde‐3‐phosphate dehydrogenase (GAPDH) was used as a housekeeping gene.

**TABLE 2:** Primer list

Gene	Forward primer: 5′‐3′	Reverse primer: 5′‐3′
Mouse BDNF	TGCAGGGGCATAGACAAAAGG	CTTATGAATCGCCAGCCAATTCTC
Mouse CD68	CCAATTCAGGGTGGAAGAAA	ATGGGTACCGTCACAACCTC
Mouse GAPDH	TGGCCTTCCGTGTTCCTAC	GAGTTGCTGTTGAAGTCG
Mouse GAP43	CTGCTGTCACTGATGCTGCT	GGTTTGGCTTCGTCTACAGC
Mouse GDNF	ATTTTATTCAAGCCACCATTA	GATACATCCACACCGTTTAGC
Mouse IL‐4	GGCTTTCCTCTTTCCCACTC	AGCCGCCATGAGAGCTAAG
Mouse IL‐10	GCTGAGACTTTCGCTCCTCTC	AGCTCCAAGGCACCTGTTC
Mouse IL‐13	TCCAATTGCAATGCCATCTA	TGGGCTACTTCGATTTTGGT
Mouse iNOS	GGCCAGCCTGTGAGACCTTT	TTGGAAGTGAAGCGTTTCG
Mouse NGF	CAAGGACGCAGCTTTCTATACTG	CTTCAGGGACAGAGTCTCCTTCT
Mouse SCG‐10	TTCTCGTGGAGTGTGTGCTTCACT	TAGCTTTGTGTTTGTGTTCGGCCC

### 2.5 Bone marrow‐derived macrophage (BMDM) primary culture

Mice were euthanized with pentobarbital diluted in saline (1:1) and cleaned with ethanol 70%. Femur and tibial bones were dissected, bone epiphyses removed, and bone marrows flushed with chilled PBS using a 10‐ml syringe and 25G needle. Cells were centrifuged at 500 RCF for 10 min, to be later cultured in 100 ml Petri plates with DMEM/F12 medium that contained 10% fetal bovine serum, 1% penicillin–streptomycin, and l‐glutamine. Macrophage colony‐stimulating factor (M‐CSF) was added at 10 ng/ml. Medium was replaced every 3 days with the correspondent addition of M‐CSF. At 8 days in vitro (div), adherent cells were reseeded in 6‐well plate in a density of 1.6 × 10^6^ cells/well with the medium mentioned above, but without the differentiating factor. Cells were treated at 10 div with vehicle containing DMSO or JQ1 1,000 nM for 6 hr (*n* = 3 independent experiments). Then, conditioned medium was harvested and snap‐frozen with liquid nitrogen.

Media from treated‐BMDM cells were filtered through 10 kDa ultrafiltration filters (Merck Millipore). Samples were centrifuged at 4°C and 10 000× *g* for 1 hr. A concentrated medium volume of 50 μl was obtained from each 1 ml medium sample, thus concentrated 20×. Concentrated media were snap‐frozen in liquid nitrogen.

### 2.6 DRG explant culture with or without BMDM‐conditioned media

Mice were euthanized with pentobarbital diluted in saline (1:1) and washed in 70% ethanol. All DRGs were extracted using sterilized surgery tools and preserved in cold Gey’s balanced solution with 0.6% glucose. DRG roots were removed and nervous tissue was exposed. DRGs were placed in a 24‐well plate between two droplets of collagen mixture in Neurobasal‐A medium with 1% penicillin–streptomycin, 1x B‐27, 0.5% glucose, 0.5% glutamax, and murine β‐NGF (100 ng/ml, Peprotech). The collagen mixture used for the culture consisted in 446.43 μl of rat collagen type 1 (Corning), 50 μl of MEM 10X medium, 2 μl of 7.5% sodium bicarbonate (Gibco), and 1.57 μl of PBS. Either JQ1 (500 or 1,000 nM) or DMSO was added into the collagen and into the medium. The culture was maintained for 2 div before fixation and immunohistochemical analysis.

For DRG explant culture with BMDM‐conditioned medium, the procedure was the same except for the preparation of the collagen mixture. In this case, half of the MEM 10x medium was substituted with conditioned medium and the amount of sodium bicarbonate added to the mixture was 8 μl. Provided that JQ1 is a small compound that is filtered during medium concentration, JQ1 or DMSO was also added to collagen to obtain a 500 nM concentration. This procedure resulted in four experimental culture groups: DMSO‐treated DRGs with conditioned medium from DMSO‐treated macrophages (D + mD), DMSO‐treated DRGs with conditioned medium from JQ1‐treated macrophages (D + mJQ1), JQ1‐treated DRGs with conditioned medium from DMSO‐treated macrophages (JQ1 + mD), and JQ1‐treated DRGs with conditioned medium from JQ1‐treated macrophages (JQ1 + mJQ1) (*n* = 3 independent experiments). The cultures were also maintained 2 div before fixation.

### 2.7 Determination of neurite outgrowth in DRG explants

After 2 div, DRG explants were fixed for 30 min with PFA 4%. Then, samples underwent several washes with TBS‐0.1% Tween before being incubated for an hour in citrate buffer (pH = 6.4) heated until boiling temperature, and washed again in TBS‐0.1% Tween. DRGs were incubated in increasing concentrations of methanol (50%, 70%, and 100%), washed in TBS‐0.1% Tween and incubated for 48 hr at 4°C in TBS‐0.3% Triton, 5% normal donkey serum, and anti‐H‐Neurofilament antibody (Table 1). DRGs were washed with TBS‐0.1% Tween and incubated overnight at 4°C with TBS‐0.3% Triton, 5% normal donkey serum, and Alexa fluor 594 Donkey anti‐mouse (Table 1). Samples were washed, dried, and mounted with Fluoromont.

DRG samples were observed under a fluorescence microscope (Olympus BX51). Images of overlapping territories of each DRG were taken with 20X objective, the microscope camera (Olympus PD37) and CellSens Entry Olympus software (https://www.selectscience.net/products/cellsens‐entry‐‐‐microscopy‐imaging‐software/?prodID=172461, RRID:SCR_014551). DRG images were reconstructed with Photoshop. Maximum neurite length and the number of neurites of different lengths were measured with the Image J plugin Neurite J (Torres‐Espin et al., 2014). The software provided the number of neurite intersections every 25 μm, and the longest neurite value for each DRG. Number of analyzed samples per group was between five and seven DRGs in the BET inhibition study, and between seven and 12 in the conditioned medium study, obtained from three independent cultures in both cases. The analysis of neurite outgrowth was performed by a blinded examiner.

### 2.8 Cell viability assay from dissociated DRGs' culture

Mice were euthanized with pentobarbital and saline mixture (1:1). All dorsal root ganglia were extracted with sterilized tools and kept in cold Gey’s balanced solution with 2% glucose. DRG roots were cut to disclose nervous tissue. Then, cleaned ganglia underwent an enzymatic digestion with trypsin 1x, collagenase 1x, and DNase (1 mg/ml) diluted in Hank’s Balanced Salt Solution (HBSS, Gibco) for 20 min at 37°C. Next, enzymatic digestion was arrested with DMEM/F12 medium containing 10% FBS, 1% penicillin and streptomycin, and l‐glutamine, before proceeding with the mechanical digestion. Cells were then filtered within a 70‐μm sterile filter to remove myelin fragments. Dissociated ganglia underwent a centrifugation at 900 rpm for 7 min and neurons were counted with a Neubauer chamber. A total of eight wells were seeded per animal in a 96‐multiwell plate pretreated with Poly‐d‐lysine (0.01 mg/ml) and laminin (2 μg/ml). Each well held between 5000 and 6000 neurons that were maintained in Neurobasal‐A medium with 2% B‐27, 2% glucose at 30%, 1% glutamine, and 1% penicillin and streptomycin. After 2 hr, medium was replaced.

At 2 div, cells were treated in duplicates with vehicle containing DMSO, JQ1 500 nM, JQ1 1000 nM, or cisplatin 5 μg, used as a positive control. At 4 div, an MTT (3‐[4,5‐dimethylthiazol‐2‐yl]‐2,5‐diphenyltetrazolium bromide) assay was performed to determine cell viability. For this purpose, media was replaced with media containing 0.15 mg/ml MTT. Next, the culture was maintained at 37°C for 2 hr, to finally obtain cells lysed with DMSO. Absorbance was read out through a spectrophotometer (Bio‐tek) at 560 nm and 600 nm wavelength using KC Junior software. Readings were normalized against control to obtain the percentage of survival. In total, there were five independent cultures.

### 2.9 Western blot

Protein samples for western blot analysis were obtained from DRG explant cultures treated with conditioned medium from DMSO‐ or JQ1‐treated macrophages. At 2 div, collagen layers encapsulating the ganglia were removed using forceps (Dumont #5). DRGs were collected in a cryovial according to the treatment and were immediately snap‐frozen in liquid nitrogen. Next, DRGs were homogenized with modified RIPA buffer with protease (100 X) (Sigma) and phosphatase (20X) (Roche) inhibitors. Samples underwent sonication and centrifugation at 14,000 rpm for 20 min at 4°C. Protein concentration of the supernatant was determined with a bicinchoninic acid assay (Pierce). SDS‐PAGE was conducted in a 7.5% acrylamide gel, where 20 μg of protein was loaded from each sample. Proteins were transferred to a PVDF membrane, that was blocked in 3% BSA prior to an overnight incubation with the appropriate primary antibodies. Several washings with TBS‐Tween 0.1% were performed and the correspondent secondary antibodies incubated during 2 hr at room temperature. Finally, membranes underwent multiple washes with TBS‐Tween 0.1% and TBS, before image acquisition with Bio‐Rad Chemidoc XRS Gel Imaging System (https://www.bio‐rad.com/en‐us/product/chemidoc‐xrs‐system?ID=NINJHRKG4, RRID:SCR_019690). Quantification was then assessed using the ImageLab software (http://www.bio‐rad.com/en‐us/sku/1709690‐image‐lab‐software, RRID:SCR_014210). Protein levels were analyzed considering the relative intensities of the bands of the phosphorylated protein forms, which were later compared to total non‐phosphorylated protein. Then, results were normalized to control. Primary and secondary antibodies used for western blot are in Table 1. There were three blots per protein analysis.

### 2.10 Statistical analysis

Data were expressed as mean ± standard deviation (SD) as minimum to maximum in Box and Whisker plots. Data distribution and outliers were determined using the GraphPad Prism software. Results were analyzed with GraphPad Prism 7 software, and significant differences considered when *p* < 0.05. For electrophysiological tests, CMAP values were analyzed by two‐way ANOVA followed by Tukey's correction for multiple comparisons, with day and amplitude or latency as factors. The CNAP results were compared with an unpaired *t* test since only a final value was recorded. Histological data of pad reinnervation were analyzed by unpaired *t* test. For statistical analysis of mRNA expression, maximum neurite length of DRG explants treated with increasing doses of JQ1, and cell viability assay, one‐way ANOVA followed by a multiple comparison test with Tukey’s correction was performed, since only the level of the mRNA, the number of neurites. and % of viable cells were analyzed within the different conditions. The number of neurites at variable distances was analyzed through a two‐way ANOVA followed by Sidak’s correction for multiple comparisons, using the distance growth and treatments as factors. Maximum neurite length for the conditioned medium experiment was also analyzed using a two‐way ANOVA followed by Sidak's correction, in which conditioned media and treatment were factors. For WB analysis, a paired *t* test was conducted, to compare the level of the protein between treatments.

## 3 RESULTS

### 3.1 Delayed treatment with JQ1 promotes enhanced expression of anti‐inflammatory cytokines and the axonal growth marker GAP43 after sciatic nerve crush

**FIGURE 1:**
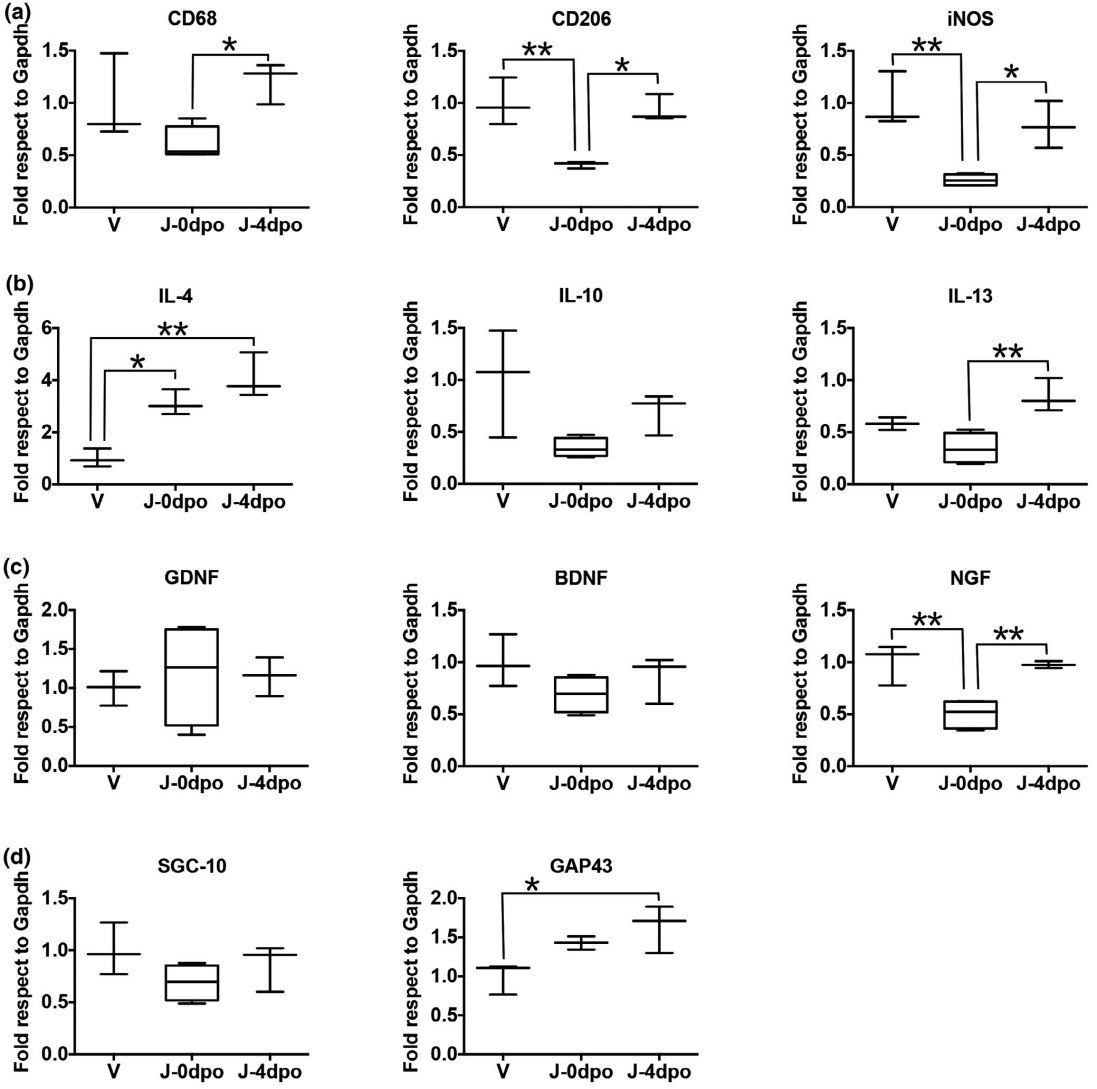
Delayed BET inhibition with JQ1 increases anti‐inflammatory cytokine and GAP43 transcription in vivo. (a) Early treatment with JQ1 at 0 dpo significantly decreases macrophage infiltration detected with CD68. Neither of the treatments alters M1/M2 polarity as detected with iNOS (M1) and CD206 (M2). (b) Delayed BET inhibition increases the transcription of IL‐4 and IL‐13 anti‐inflammatory cytokines. (c) Immediate administration of JQ1 at 0 dpo decreases NGF mRNA expression. (d) BET inhibition at 4 dpo increases GAP43 transcription, while not altering the mRNA levels of SGC‐10. *p < 0.05 and **p < 0.01 as calculated by one‐way ANOVA followed with a multiple comparison with Tukey’s correction. Data are shown as minimum to maximum box and whisker graphs

Since BET protein inhibition by JQ1 alters macrophage transcriptional state (Nicodeme et al., 2010; Sanchez‐Ventura et al., 2019) and macrophage infiltration after PNI occurs early after injury, we compared the effect between an early and a delayed treatment with JQ1 in sciatic nerve (Figure 1a). We observed a reduced expression of the macrophage marker *Cluster of differentiation 68* (CD68) when the treatment started at 0 dpo, indicating a reduction on macrophage infiltration (*F*_2,7_ = 4.637, *p* = 0.052). The expression of *inducible nitric oxide synthase* (iNOS) and CD206, which correspond to M1 and M2 macrophage markers, respectively, was also reduced within this treatment, probably due also to a lessened macrophage infiltration (iNOS, *F*_2,6_ = 14.170, *p* = 0.004) (CD206, *F*_2,6_ = 13.660, *p* = 0.006). However, when the treatment started at 4 dpo, we did not observe changes in CD68, iNOS, or CD206 mRNA expression compared to vehicle‐treated animals, indicating that the delayed treatment allowed macrophages to infiltrate, and that JQ1 does not affect M1/M2 macrophage polarization. IL‐4 and IL‐13 anti‐inflammatory cytokine expression was statistically enhanced within animals treated with JQ1 from 4 dpo (IL‐4, *F*_2,6_ = 20.380, *p* = 0.002) (IL‐13, *F*_2,6_ = 10.520, *p* = 0.011), whereas IL‐10 was not altered (*F*_2,7_ = 4.023, *p* = 0.069) (Figure 1b). A mild increase of IL‐4 also was observed when the treatment started at 0 dpo. We further studied the effects of JQ1 in neurotrophic factor expression. We did not observe changes of *glial‐derived neurotrophic factor* (GDNF) (*F*_2,7_ = 0.137, *p* = 0.874) and *brain‐derived neurotrophic factor* (BDNF) (*F*_2,7_ = 1.833, *p* = 0.229) expression, whereas we found a reduction of *nerve growth factor* (NGF) with the treatment starting at 0 dpo (*F*_2,7_ = 14.400, *p* = 0.003), probably due to the reduced infiltration of macrophages.

We then wanted to ascertain if the transcriptional alterations promoted by JQ1 produced an effect on axonal growth markers expression (Figure 1c). We did not observe an alteration in the *superior cervical ganglion‐10 protein* (SGC‐10) (*F*_2,6_ = 1.833, *p* = 0.229), whereas we found an enhanced expression of the *Growth associated protein 43* (GAP43) when the treatment started at 4 dpo (*F*_2,6_ = 6.698, *p* = 0.0270). Therefore, we planned to further study the effects of axonal regeneration in vivo by a delayed treatment with JQ1.

### 3.2 BET protein inhibition does not enhance nerve regeneration after sciatic nerve crush

**FIGURE 2:**
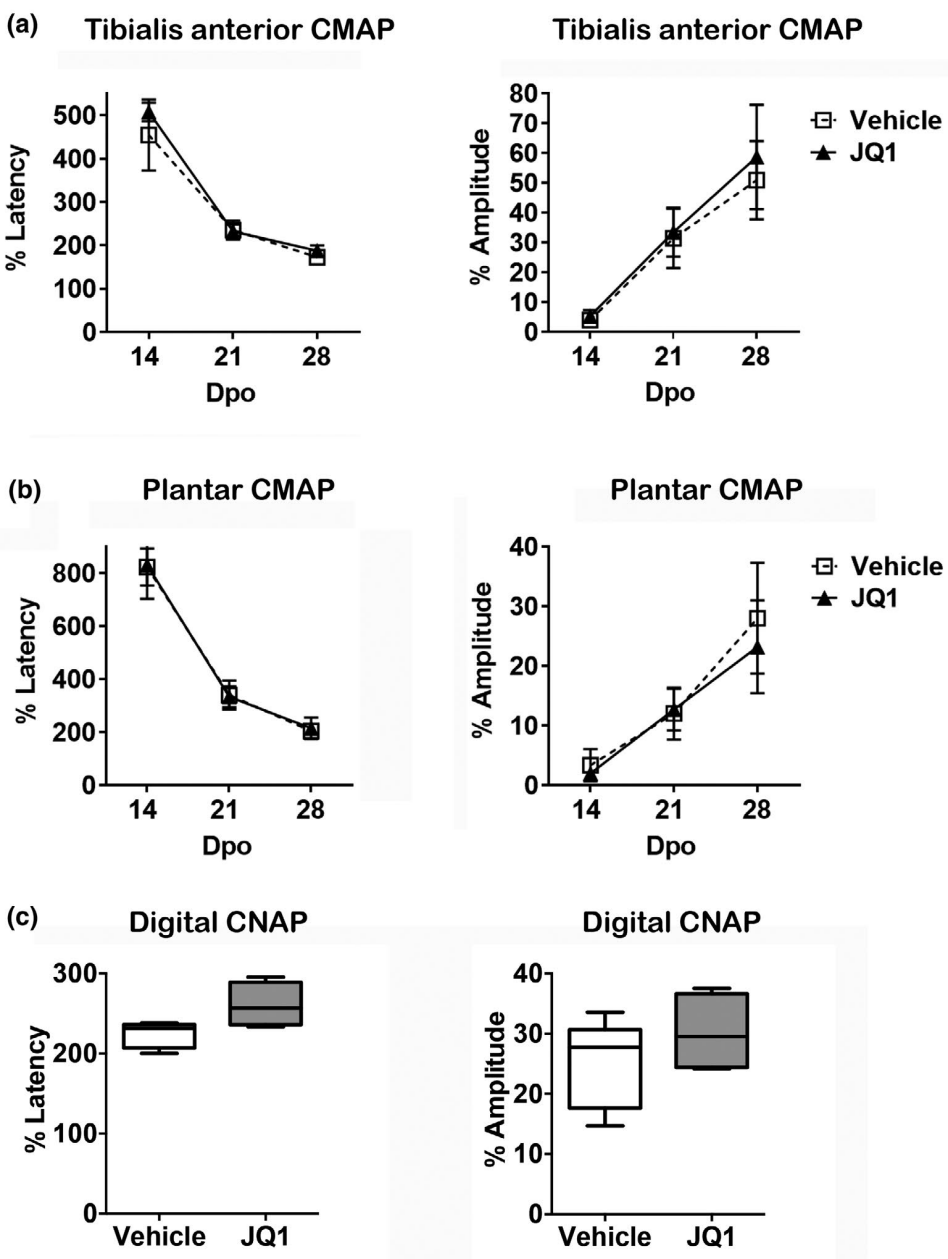
BET proteins do not affect nerve regeneration after sciatic nerve crush assessed by electrophysiological tests. No significant differences were found between BET‐inhibited animals (JQ1) and control animals (DMSO) in the electrophysiological recordings of the latency and amplitude of the CMAP of tibialis anterior muscle (a), CMAP of plantar muscle (b), and CNAP of the digital nerve (c). Data shown as mean ± SD and minimum to maximum in box and whisker graphs

The analysis of functional and histological outcome was performed in mice with delayed treatment of vehicle and JQ1. Motor nerve conduction tests determine muscle reinnervation, as recorded CMAPs, by regenerated axons after injury. CMAPs were first recorded in the tibialis anterior and plantar muscles at 14 dpo, being of small amplitude and increased latency (Figure 2). At later time points, the latency time decreased indicating axonal enlargement and remyelination after reinnervating the muscle fibers. No differences were found between vehicle and JQ1‐treated animals (*F*_2,14_ = 1.380, *p* = 0.280 for tibialis anterior and *F*_2,14_ = 0.057, *p* = 0.945 for plantar muscle). Regarding the amplitude of the CMAP, which determines the amount of innervated muscle fibers, as indicator of nerve regeneration, at 14 dpo the M amplitude was very low in both muscles, around 5% of control values in the tibialis anterior muscle (Figure 2a) and 2.5% in the plantar muscle (Figure 2b). At 21 and 28 dpo, the CMAP amplitude increased in both muscles recorded. However, there were not significant differences between JQ1‐treated and vehicle‐treated groups (*F*_2,14_ = 0.354, *p* = 0.708 for tibialis anterior and *F*_2,14_ = 0.596, *p* = 0.565 for plantar muscle).

For the sensory nerve conduction test, CNAPs of the digital nerve were recorded only at 28 dpo. The CNAP latency was increased in the two groups of mice (*t*_7_ = 2.516, *p* = 0.040). Regarding CNAP amplitude, although JQ1‐treated group showed a tendency to be higher (30%) compared to the vehicle‐treated group (25%), the difference was not statistically significant (Figure 2c) (*t*_7_ = 1.126, *p* = 0.297).

**FIGURE 3:**
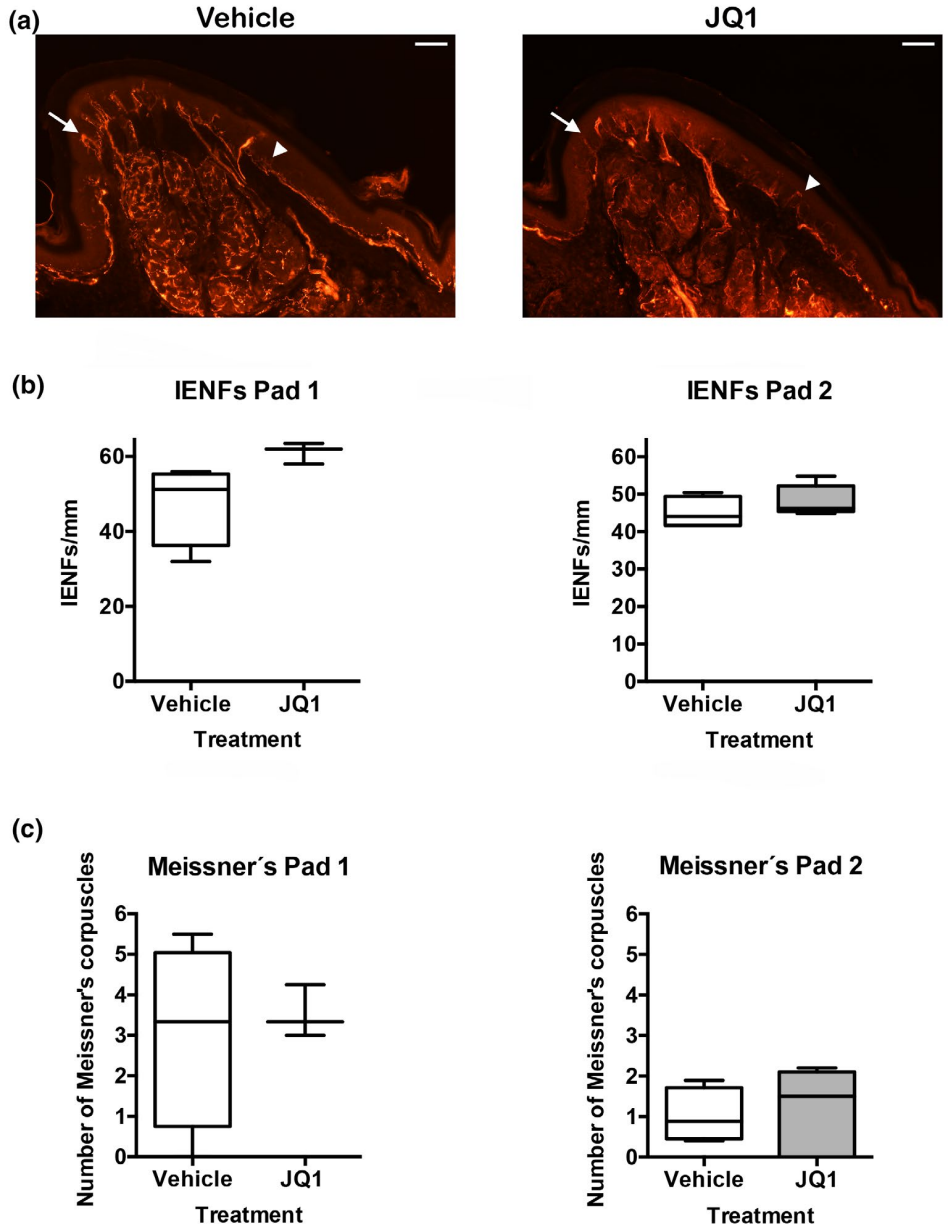
BET protein inhibition does not modify sensory reinnervation after sciatic nerve crush. (a) Microphotographs of hind paw pads at ×10 magnification of vehicle and JQ1‐treated animals immunolabeled against PGP9.5. Arrow tips point IENFs, whereas arrows exhibit the location of Meissner’s corpuscles. Scale bar = 100 μm. (b) No significant differences were found in IENFs density of vehicle and JQ1‐treated mice in medial pad 1 and lateral pad 2. (c) No significant differences were found in the number of Meissner’s corpuscles of vehicle and JQ1‐treated animals in pad 1 and pad 2. Data shown as minimum to maximum in box and whisker graphs

To further study the effects of BET inhibition on axonal regeneration, we analyzed skin reinnervation of the hind paw. Regarding IENF density, JQ1‐treated animals showed a tendency of higher number of IENFs/mm (Figure 3b), compared to vehicle‐treated animals. However, no statistical differences were found. This was observed in medial Pads 1 (Vehicle: 47.58 ± 5.39**;** JQ1: 61.17 ± 1.64) (*t*_5_ = 2.082, *p* = 0.092), as well as in lateral Pads 2 (Vehicle: 45.05 ± 2.10; JQ1: 48.28 ± 1.81) (*t*_7_ = 1.166, *p* = 0.280). Regarding the number of Meissner’s corpuscles (Figure 3c), no significant differences were found between treatments in Pads 1 (Vehicle: 3.04 ± 1.14; JQ1: 3.53 ± 0.37) (*t*_5_ = 0.350, *p* = 0.741) or Pads 2 (Vehicle: 1.02 ± 0.33; JQ1: 1.14 ± 0.47) (*t*_7_ = 0.202, *p* = 0.845).

Therefore, the results of in vivo tests showed that JQ1 administration did not have any significant effect on axonal regeneration in the mice.

### 3.3 BET protein inhibition reduces DRG neurite outgrowth

We analyzed the effects of BET protein inhibition on neurite outgrowth in DRG explants ex vivo, by adding to the medium variable doses of JQ1 or DMSO as control. Two parameters were analyzed in this study: maximum neurite length and number of neurites of different lengths.

**FIGURE 4:**
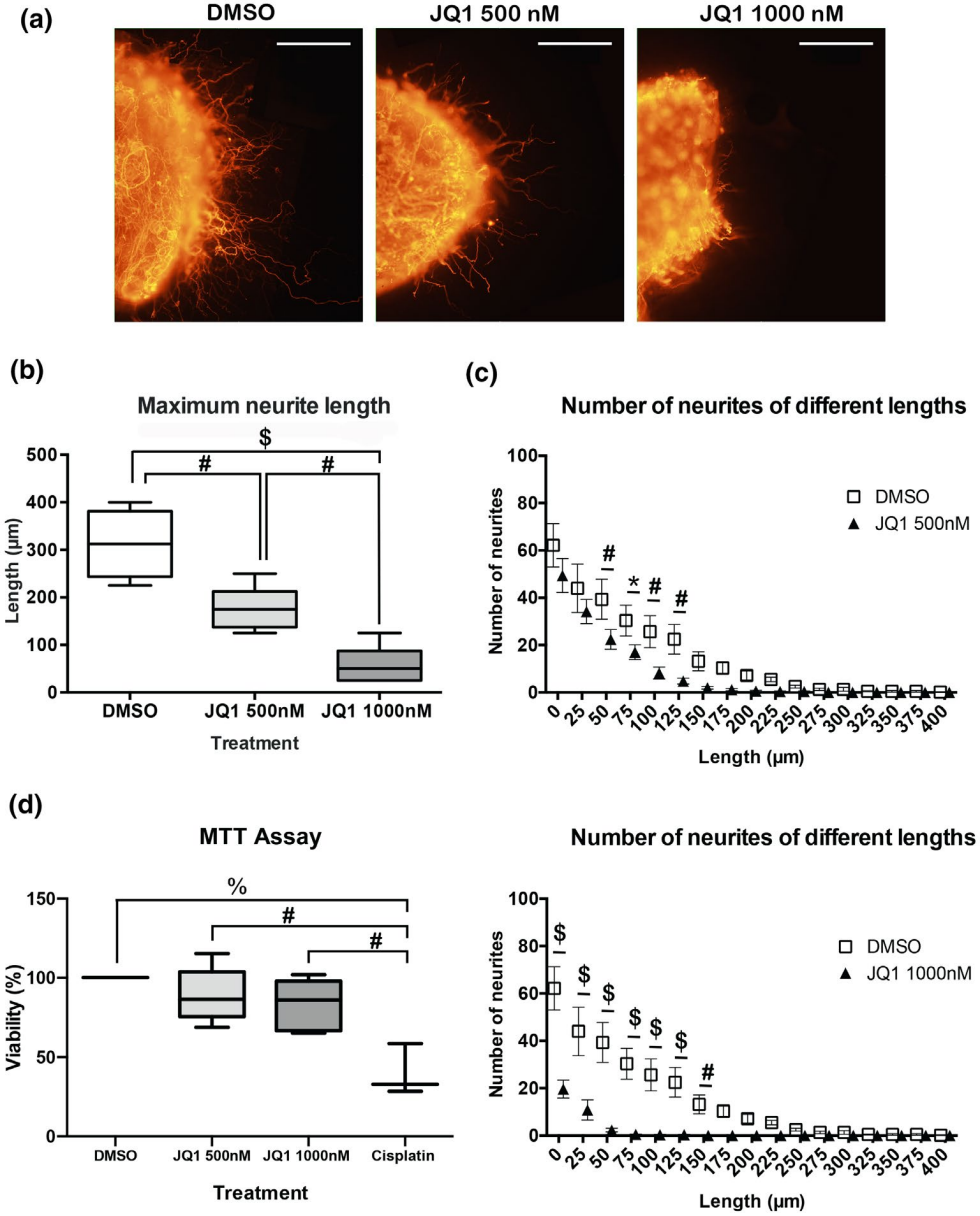
BET protein inhibition reduces DRG neurite outgrowth ex vivo. (a) Representative images from DRG explants at ×20 magnification show that BET protein inhibition decreases neurite outgrowth proportionally to JQ1 concentration. Scale bar = 100 μm. (b) BET inhibition with JQ1 reduces maximum neurite length. (c) JQ1 at 500 nM and 1000 nM significantly diminish the number of neurites of different lengths when compared to DMSO‐treated ganglia. (d) The different concentrations of JQ1 used in vitro do not produce cell death. *p < 0.05, ^#^ *p* < 0.01, ^%^ *p* < 0.001, and ^$^ *p* < 0.0001 as calculated by one‐way ANOVA with Tukey’s multiple comparison test and two‐way ANOVA analysis followed by Sidak’s correction of multiple comparisons. Data shown as mean ± SD or minimum to maximum in box and whisker graphs

DMSO‐treated DRGs had a mean maximal neurite length of 313 μm, whereas JQ1 500 nM and JQ1 1000 nM treatment led to a significant decrease on the maximum neurite length, reaching mean values of 175 μm and 58 μm, respectively (Figure 4a,b) (*F*_2,14_ = 34.610, *p* < 0.001). The number of neurites grown at different lengths showed that DRGs treated with 500 nM JQ1 had a significant decrease on the number of neurites ranging from 50 μm to 125 μm in length (Upper Figure 4c), compared to DMSO‐treated ganglia (*F*_16,340_ = 2.280, *p* = 0.004). The decrement in neurite growth was more pronounced with JQ1 at 1000 nM, showing a significant reduction on the amount of neurites from 0 μm to 150 μm compared with DMSO (Lower Figure 4c) (*F*_16,374_ = 16.840, *p* < 0.001).

Therefore, BET inhibition produced a significant decrease on DRG neurite outgrowth, that was dependent on the JQ1 concentration. To determine if this decrease was due to compound toxicity, we performed an MTT assay (Figure 4d). Results showed that 500 or 1,000 nM of JQ1 did not produce cell death, whereas the positive control cisplatin led to 60.07% of cell death (*F*_3,14_ = 12.330, *p* < 0.001).

### 3.4 BET protein‐inhibited macrophages secrete pro‐regenerative factors

Results obtained from in vivo and ex vivo data indicated contradictory effects of BET protein inhibition on axonal growth. Therefore, we hypothesized that these differences might be produced by macrophages, which infiltrate the nerve after lesion in vivo, but were missing in the DRG cultures. To assess the effects that BET‐inhibited macrophages have on neurons ex vivo, we cultured DRG explants with conditioned medium from JQ1 or DMSO‐treated macrophages added into the collagen matrix.

**FIGURE 5:**
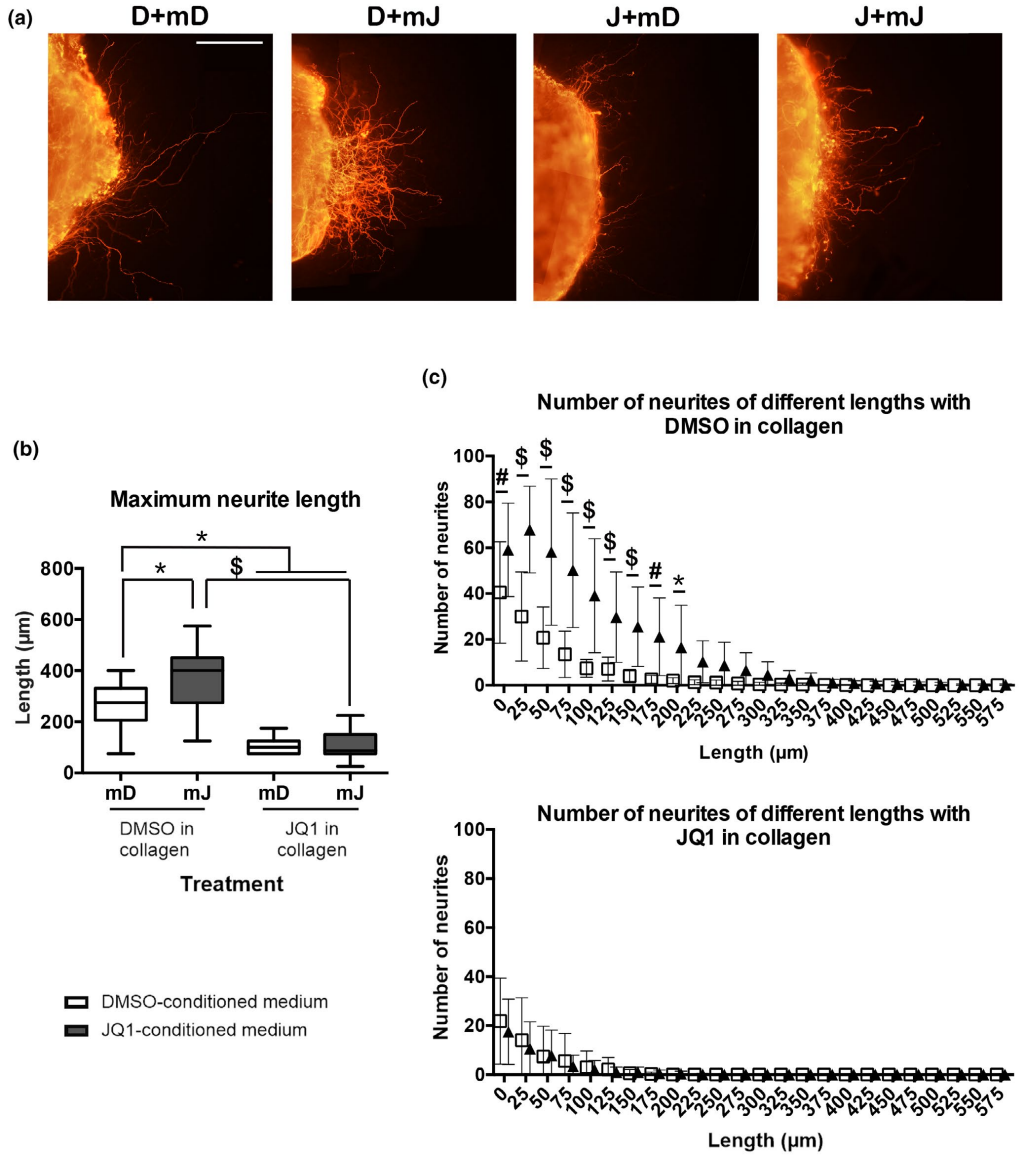
Conditioned media from BET‐inhibited macrophages enhance DRG neurite outgrowth. (a) Representative images from DRG explants at ×20 magnification show that media from BET‐inhibited macrophages enhances neuritogenesis. Scale bar = 100 μm. (b) Conditioned media from BET‐inhibited macrophages increases maximum neurite length in vitro. From left to right: DMSO in collagen + DMSO‐conditioned medium (D + mD), DMSO in collagen + JQ1‐conditioned medium (D + mJ), JQ1 in collagen + DMSO‐conditioned medium (J + mD), and JQ1 in collagen + JQ1‐conditioned medium (J + mJ). (c) Conditioned media from JQ1‐treated macrophages increases the number of neurites of different lengths when DMSO is in collagen (upper graph). No significant differences are found between treatments regarding the number of neurites of different lengths when JQ1 is in collagen (lower graph). *p < 0.05, ^#^ *p* < 0.01, and ^$^ *p* < 0.0001 as calculated by two‐way ANOVA with Sidak’s correction of multiple comparisons. Data shown as mean ± SD or minimum to maximum in box and whisker graphs

Control DRGs treated with DMSO‐conditioned medium (D + mD) had a maximum neurite length of 258 μm in average. A significant increase in neurite outgrowth was found when DRGs were treated with JQ1 conditioned medium (D + mJQ1), with a mean maximal neurite length of 370 μm (Figure 5a,b). Since conditioned media from macrophages was concentrated by filtration, this media contained 20 times less of JQ1 small compound while increased the concentration of high molecular weight molecules secreted by macrophages. Thus, we can discard any direct effect of JQ1 on the cultured neurons. Therefore, these results indicate that BET‐inhibited macrophages secrete pro‐regenerative factors that stimulate neurite outgrowth.

Then, we wanted to assess if these pro‐regenerative factors were also able to counteract the effects produced by direct BET protein inhibition on DRG neurons. For this purpose, JQ1 was added into the collagen and the medium of DRG explants. When JQ1 was administered in the collagen, it reduced neurite outgrowth, despite the conditioned media added (Figure 5a,b). Significant differences were observed between D + mD (258 μm) and J + mD (107 μm), and between D + mJ (370 μm) and J + mJ (109 μm). In addition, contrarily to what we expected, JQ1 conditioned medium did not overcome the detrimental effects of direct BET inhibition on neurons, since there were no differences between J+ mD (107 μm) and J + mJ (109 μm) (Interaction *F*_1,34_ = 2.974, *p* = 0.0937; Row factor (conditioned media) *F*_1,34_ = 3.221, *p* = 0.0816; and Column factor (treatment) *F*_1,34_ = 41.86, *p* < 0.0001) (Figure 5b). Hence, this experiment confirmed our hypothesis that cultured macrophages secreted factors that stimulated neurite growth when treated with JQ1.

To further corroborate our findings, we determined the number of neurites of different lengths. We found that DMSO‐treated DRGs with JQ1 conditioned medium (D + mJ) had a significant increase in the number of neurites ranging from 0 μm to 200 μm (Figure 5c, top), when compared to DMSO‐treated ganglia with DMSO‐conditioned medium (D + mD) (Interaction *F*_23,504_ = 8.631, *p* < 0.0001; Row factor (length) *F*_23,504_ = 48.89, *p* < 0.0001; and Column factor (treatment) *F*_1,504_ = 152.6, *p* < 0.0001). However, no significant differences were found between JQ1‐treated DRGs with DMSO‐conditioned medium (JQ1 + mD) and JQ1‐treated DRGs with JQ1‐conditioned medium (JQ1 + mJQ1) (Figure 5c, bottom) (Interaction *F*_23,312_ = 0.1894, *p* > 0.9999; Row factor (length) *F*_23,312_ = 11.91, *p* < 0.0001; and Column factor (treatment) *F*_1,312_ = 0.6133, *p* = 0.4342). These results are consistent with the maximum neurite length analysis, as it demonstrates that JQ1‐treated macrophages conditioned medium increased neurite outgrowth. Nonetheless, JQ1‐conditioned medium did not overcome the negative effects of direct BET inhibition, probably due to the high concentration of JQ1 used in the ex vivo explants.

### 3.5 Conditioned medium from BET‐inhibited macrophages induce STAT6 phosphorylation in DRG explants

**FIGURE 6:**
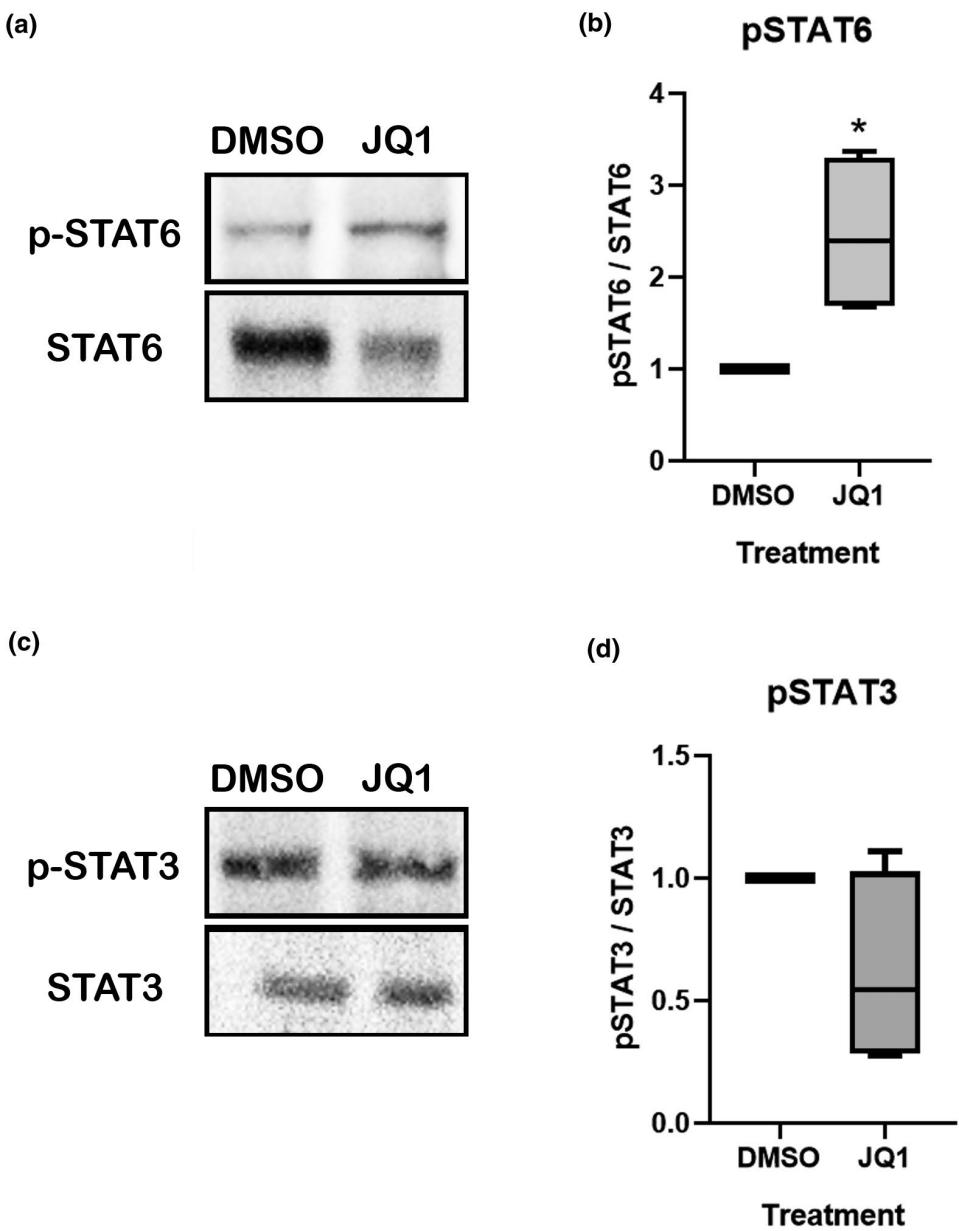
STAT6 phosphorylation in DRG explants is produced by conditioned media from BET‐inhibited macrophages. (a) Representative images obtained from western blots against p‐STAT6 and STAT6 of DRGs treated with conditioned media from macrophages treated with JQ1 or vehicle. (b) Quantification of (a) showing that DRGs treated with conditioned media from BET‐inhibited macrophages increase STAT6 phosphorylation relative to STAT6, compared to ganglia treated with conditioned media from DMSO‐treated macrophages (DMSO). (c) Representative images from western blots against p‐STAT3 and STAT3 of DRGs treated with conditioned media from macrophages treated with JQ1 or vehicle. (d) Quantification of (c) showing that there were no significant differences regarding the protein levels of p‐STAT3 relative to STAT3 of DRGs treated with conditioned media from DMSO‐treated macrophages and BET‐inhibited macrophages. *p < 0.05 as calculated by a paired *t* test. Data shown as minimum to maximum in box and whisker graphs

Our previous experimental study described that BET‐inhibited macrophages increase anti‐inflammatory cytokine expression of IL‐4, IL‐10, and IL‐13 (Sanchez‐Ventura et al., 2019). Moreover, former qPCR analysis demonstrates that JQ1 treatment at 4 dpo after sciatic nerve crush increases IL‐4 and IL‐13 cytokine transcription. As experimental evidence points that anti‐inflammatory cytokines promote neurite outgrowth through acting on JAK/STAT pathways (Vidal et al., 2013), we aimed to determine the activation state of these pathways. For this purpose, DRG explants were treated with conditioned media from JQ1 or DMSO‐treated macrophages. We found that ganglia treated with conditioned medium from BET‐inhibited macrophages displayed a significant increase in the phosphorylation of STAT6 (Figure 6a,b, Figure S1), being 2.46 times higher than in DRGs treated with conditioned medium from DMSO‐treated macrophages (*t*_3_ = 3.278, *p* = 0.046). However, no significant differences between treatments were found regarding the activation of STAT3 pathway (Figure 6c,d, Figure S1) (*t*_3_ = 1.903, *p* = 0.153). Thus, conditioned medium from BET‐inhibited macrophages led to an increased phosphorylation of STAT6, probably through an enhanced release of IL‐4 and IL‐13 anti‐inflammatory cytokines.

## 4 DISCUSSION

In the present study we have analyzed the effects of BET inhibition on nerve regeneration in vivo and neurite outgrowth ex vivo. Overall, BET inhibition after sciatic nerve crush injury in mice did not produce any effect on axonal regeneration. In contrast, direct inhibition of BET proteins in neurons ex vivo produced a decrease in neuritogenesis. However, BET‐inhibited macrophages secrete pro‐regenerative factors that promoted neurite outgrowth in DRG cultures, at least by enhancing the STAT6 pathway. Thus, specific targeting of BET proteins in macrophages may be needed to efficiently enhance axonal regeneration and functional recovery after PNI.

The effects of JQ1 treatment on axonal growth were first analyzed after sciatic nerve crush injury in mice. Results of the mRNA expression analysis demonstrated a reduced infiltration of macrophages when JQ1 treatment started at 2 hr postinjury, whereas a delayed treatment did not. In addition, an enhanced expression of IL‐4 and IL‐13 and GAP43 was found with the delayed treatment of JQ1. It is known that BET protein inhibition reduces chemokine expression in macrophages (Nicodeme et al., 2010; Sanchez‐Ventura et al., 2019), which are essential for macrophage infiltration. Thus, we hypothesized that an early treatment with JQ1 would reduce macrophage infiltration, negatively affecting the development of Wallerian degeneration and consequently hindering future outcome. Therefore, we used a delayed treatment with JQ1 since it allows an early macrophage infiltration, which may be useful for further experiments. However, electrophysiological and histological results showed that the delayed treatment with JQ1 did not have any effect on sensory or motor reinnervation after injury. Mice were treated with 30 mg/kg/24 hr, which is the same dose we previously reported to have beneficial effects after spinal cord injury (Sanchez‐Ventura et al., 2019). Besides, increasing doses of JQ1 could not be used since long‐term administration of higher doses of JQ1 produce a reduction of body weight and eventual death of mice (data not shown). Further, it has been reported by other authors that doses over 50 mg/kg led to detrimental side effects on mice (Korb et al., 2015; Matzuk et al., 2012). Since no changes on nerve regeneration were observed in mice, to better decipher the action of BET proteins on axonal regeneration in neurons, we used DRG ex vivo cultures.

We observed that JQ1 reduced neurite growth when interacting directly with neurons in culture. The mechanisms producing neurite outgrowth inhibition are not known. Cell viability assay demonstrated that JQ1 was not toxic at the used concentrations, confirming the results of other authors in human mesenchymal stem cells and neurons (Bakshi et al., 2018; Li et al., 2020). It has been previously reported that treatment with JQ1 on cortical neurons produce gene repression of immediate early genes such as Arc and Fos Nr4a1 in response to BDNF external stimulation (Korb et al., 2015). Thus, it seems appropriate to hypothesize that JQ1 prevents the expression of genes associated with neurite growth in neurons. However, an exhaustive study remains to be performed to determine which neuronal genes are directly affected by BET inhibition. In addition, several authors have reported that JQ1 treatment leads to a decrease in mTOR phosphorylation (Jang et al., 2017; Li et al., 2019; Li et al., 2020). Therefore, since it has been described that mTOR activation leads to neurite outgrowth and peripheral nerve regeneration (Abe et al., 2010; Chen, Lu, et al., 2016; Liu et al., 2019), it is also possible that BET inhibition in neurons could reduce the intrinsic regenerative capacity of neurons by decreasing the activation of mTOR pathway. Nonetheless, deeper research should be performed to confirm the former hypothesis.

Further studies demonstrated that the discrepancy of the JQ1 effect on axonal growth between the in vivo and ex vivo experimental settings could be accounted to the effect of BET inhibition in macrophages in vivo. Conditioned medium from JQ1‐treated macrophages enhanced neurite outgrowth in DRG neurons, thus indicating that BET‐inhibited macrophages secrete pro‐regenerative factors that induce neuritogenesis. We have previously shown that treatment with JQ1 to activated macrophages reduced the expression of pro‐inflammatory cytokines, such as IL‐6, IL‐1b, and TNFα, and promoted the expression of anti‐inflammatory cytokines IL‐4, IL‐10, and IL‐13 (Sanchez‐Ventura et al., 2019). In this study, we further corroborated an enhancement of the expression of IL‐4 and IL‐13 after crush injury with JQ1. Importantly, anti‐inflammatory cytokines, secreted in the denervated distal nerve stump, have a clear role increasing the capacity of proximal axons to cross the lesion zone. It is known that animals lacking IL‐10 regenerate worse than WT animals (Siqueira Mietto et al., 2015), and that the administration of IL‐4 and IL‐10 enhance axonal regeneration (Atkins et al., 2007; Vidal et al., 2013). Therefore, our results suggest that BET inhibition leads to an increase of anti‐inflammatory cytokines that compensates the detrimental effects that JQ1 has on neurons in vivo by activating pathways able to increase axonal regeneration. In the ex vivo studies, conditioned medium from JQ1‐treated macrophages was not able to overcome the inhibitory signaling of JQ1 present in the collagen matrix embedding the DRG, thus acting directly on neurons. We hypothesize that within these cultures, JQ1 is in higher concentrations than in the in vivo study in mice, producing a stronger effect on neuronal BET proteins in the culture than in vivo.

To determine if cytokines are the potential mediators promoting neurite growth of DRG explants, STAT3 and STAT6 phosphorylation was assessed. We did not observe any changes on STAT3 phosphorylation in DRG samples treated with conditioned media from BET‐inhibited macrophages. STAT3 is activated through IL‐6 and IL‐10. In fact, IL‐6 and IL‐10 have been reported to produce neuronal regeneration through phosphorylation and concomitant activation of STAT3 (Chen, Lin, et al., 2016; Vidal et al., 2013). However, since our previous studies demonstrated that JQ1 reduces the expression of IL‐6, which may compensate for the IL‐10‐enhanced expression (Sanchez‐Ventura et al., 2019), we did not observe an effect on this pathway to explain the enhanced neuritogenesis. In contrast, we found an enhanced phosphorylation of STAT6 in DRG samples treated with conditioned media from BET‐inhibited macrophages. The best known activators of STAT6 are IL‐13 and IL‐4 (Mori et al., 2016) which are, in turn, strongly induced by STAT6 itself (Czimmerer et al., 2018). In fact, we previously found that BET‐inhibited macrophages secrete these cytokines (Sanchez‐Ventura et al., 2019), and we confirmed these results after a crush injury in sciatic nerve. Since there are previous reports showing that these cytokines promote axonal regeneration (Vidal et al., 2013), the enhanced neuritogenesis in DRG explants observed by the secreted factors from BET‐inhibited macrophages could be attributed, at least in part, to this pathway. It should be also considered that macrophages have two variants of IL‐4 receptor (IL‐4R) that are able to induce STAT6 phosphorylation. Type I IL‐4R can also lead to IRS‐2 phosphorylation and consequently to the activation of PIP3/Akt and Erk pathways (Czimmerer et al., 2018), which are also related to axonal regeneration (Hausott & Klimaschewski, 2019; Saijilafu et al., 2013). Therefore, further studies should be performed, to also analyze the implication of the PIP3/Akt and ERK pathways on the neural outgrowth induced by BET‐inhibited macrophages.

Finally, it should be also pointed out that we have only used females in this study, which may be a limitation for deciphering potential sex differences. Since differential regenerative capacities between males and females is controversial (Kovacic et al., 2004; Wood et al., 2012), a further study with male mice may be performed to obtain a complete vision of the results obtained.

In conclusion, this study demonstrates that BET protein inhibition in macrophages provides a suitable condition to enhance axonal outgrowth, and brings insight on the relevance of the anti‐inflammatory cytokines IL‐13 and IL‐4 and the STAT6 pathway in axonal regeneration. Thus, BET proteins are an effective target to improve axonal regeneration. However, a specific targeting of BET inhibition in macrophages would be needed to efficiently enhance functional recovery after PNI.

### DECLARATION OF TRANSPARENCY

The authors, reviewers and editors affirm that in accordance to the policies set by the *Journal of Neuroscience Research*, this manuscript presents an accurate and transparent account of the study being reported and that all critical details describing the methods and results are present.

## CONFLICT OF INTEREST

The authors declare that they have no conflict of interest.

## AUTHOR CONTRIBUTIONS

GP and CP performed methodology, investigation and formal analysis. GP, XN and CP performed writing and manuscript preparation. XN and CP did funding acquisition. CP performed study conception and supervision.

### PEER REVIEW

The peer review history for this article is available at https://publons.com/publon/10.1002/jnr.25036.

## Supporting information


**FIGURE S1** Uncropped images of WB from DRGs treated with media from DMSO‐ (marked as V) and JQ1‐treated macrophages (referred as J). (a) Pictures of samples from the first and second culture. (b) Membranes belonging to the third culture

Transparent Science Questionnaire for Authors

## Data Availability

The raw data can be found in the Universitat Autònoma de Barcelona repository, https://ddd.uab.cat/record/243396
