# Supplementary figures and images for: BET protein inhibition in macrophages enhances dorsal root ganglion neurite outgrowth in female mice
Source: J Neurosci Res. 2022 Feb 26;100(6):1331–46. doi: 10.1002/jnr.25036 (PMC9306766; doi:10.1002/jnr.25036)

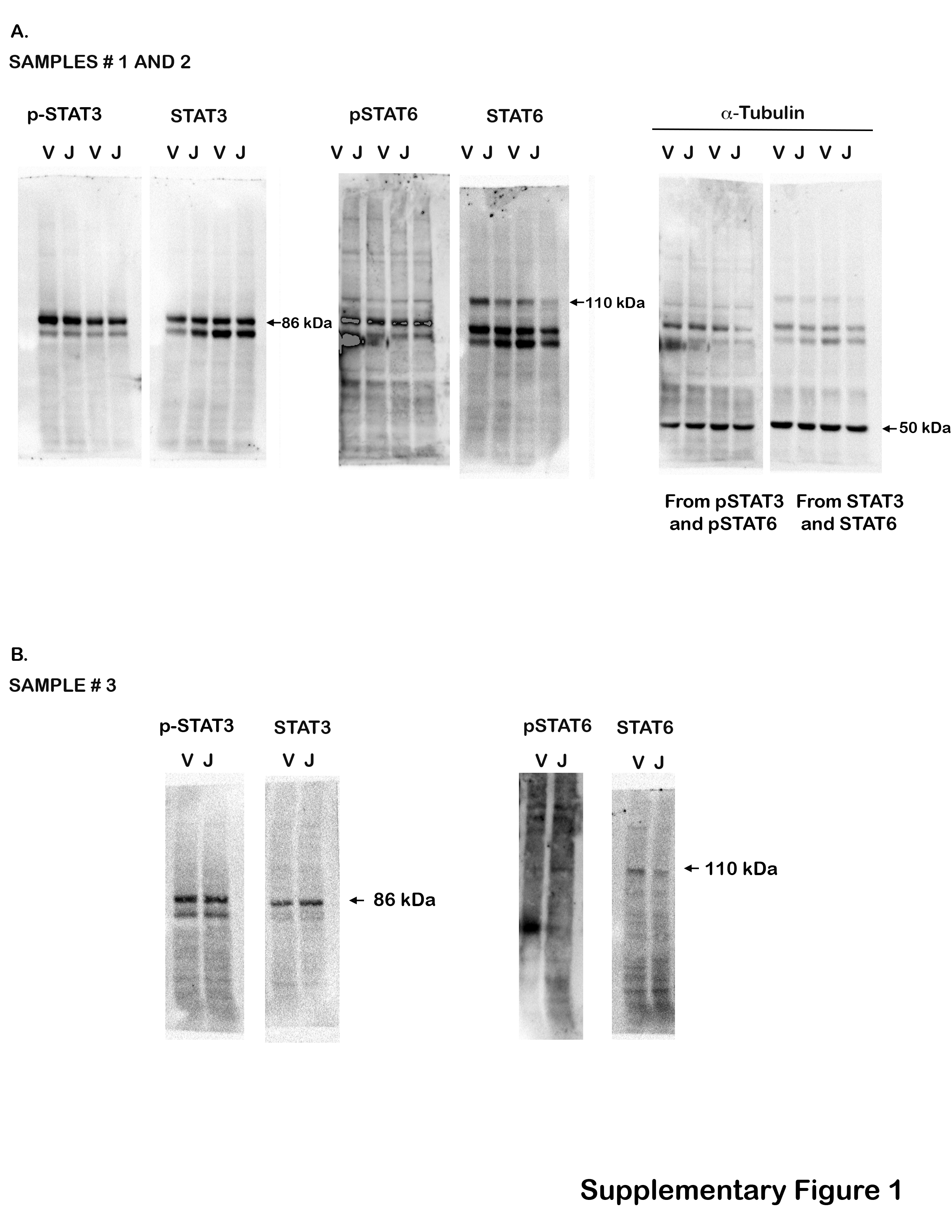

Supplement: Supplementary file 1 — FIGURE S1 Uncropped images of WB from DRGs treated with media from DMSO‐ (marked as V) and JQ1‐treated macrophages (referred as J). (a) Pictures of samples from the first and second culture. (b) Membranes belonging to the third culture [file JNR-100-1331-s001.tif]
