# Supplementary material for: BET protein inhibition in macrophages enhances dorsal root ganglion neurite outgrowth in female mice
Source: J Neurosci Res. 2022 Feb 26;100(6):1331–46. doi: 10.1002/jnr.25036 (PMC9306766; doi:10.1002/jnr.25036)
Supplement: Supplementary file 2 — Transparent Science Questionnaire for Authors [file JNR-100-1331-s002.pdf]

JOURNAL OF Neuroscience Research

Rigorous study design and transparent reporting of results are the cornerstones of science. By maximizing the information provided in a manuscript, factors that may contribute to irreproducibility will be mitigated. The *Journal of Neuroscience Research* promotes transparency in research by strongly encouraging authors to include all relevant information about their studies (see our preprint for details). To expedite reviewer monitoring of these factors, authors submitting original research articles must complete this questionnaire.

If the manuscript is accepted and all items within the checklist are present, we will include a declaration of transparency at the end of the manuscript. This declaration reads as follows:

> ***The authors, reviewers and editors affirm that in accordance to the policies set by the Journal of Neuroscience Research, this manuscript presents an accurate and transparent account of the study being reported and that all critical details describing the methods and results are present.***

To complete the checklist, fill in the right-hand column with the page and paragraph number (e.g., 'Page 3, Paragraph 2') corresponding to the checklist item. If a checklist item is not applicable to the study being reported or the authors are unable to provide that item, a reason must be supplied. Additional comments can be added at the end of the document. Upload the completed document as supplementary information for review.

Experimental and Study Design	
1. Clearly state the primary and any secondary objective or hypothesis of the study	**Page 4, paragraphs 1, 2**
2. For each experiment, the study design must include:	
a. Number of experimental and control groups	Page 5, paragraph 1, 2, 3<br>Page 6, paragraph 2<br>Page 7, paragraph 1<br>Page 8, paragraph 2<br>Page 9, paragraph 1, 4<br>Page 10 parragraph 1, 2
b. Randomization and blinding procedures and/or steps to minimize subjective bias when allocating subjects to experimental groups	Page 5, paragraph 2
c. Precise details of all procedures, including housing and husbandry are carried out in the experiment	Page 4, paragraph 3<br>Page 5, paragraph 1
d. Is sex considered as a biological variable? See Editorial for details about proper reporting	Page 19, paragraph 2
Experimental Subjects	
3. Specify the total number of subjects in each experiment, including the number of animals, sex and age in each group	
a. Explain how the number of animals were arrived at and provide details of any sample size calculation, including power analysis	Page 5, paragraph 1, 2
b. Indicate the number of independent replications of each experiment, when applicable	Materials and methods section page 4 to 10
Data Handling	
4. Indicate data collection start and stop rules:	
a. Define the criteria for data/subject inclusion and exclusion. If any outcome or condition measure used was not reported in the results section, authors must address this omission	We did not exclude values
b. Specify reasons for any discrepancy between the number of animals at the beginning and end of the study	There is no discrepancy
c. Define and explain how outliers are handled and report if data are removed prior to analysis	Page 10, paragraph 3
Statistical Analysis and Depiction of Continuous Data	
5. Provide details of the statistical methods used for each analysis	
a. State, define and justify the statistical analysis used and specify the unit of analysis for each dataset	Page 10, paragraph 3<br>Page 11, paragraph 1
b. Describe and report methods used to assess whether data met the assumptions of the statistical approach and any adjustments for multiple comparisons	Page 11, paragraph 1
c. Fully report statistics (including exact value of N, degrees of freedom, test value and exact P-value when >0.001) and we encourage the use of effect sizes and confidence intervals	In results section, pages 11 to 16
d. Disaggregated data are presented for males and females	We have only female data

JOURNAL OF Neuroscience Research

e. *Data distribution is depicted with univariate scatterplots boxplots, violin plots, or kernel density plots when presenting* ***continuous data*** *(see Editorial* Publishing Transparent and Rigorous Scientific Research)	Page 10, paragraph 3

Discussion

6. *Comment on study limitations including any potential source of bias, limitations to the animal model, imprecisions associated with the results, and the inability for any reason to study possible sex influences where they may exist.*	Page 19, paragraph 2
7. *Comment on possible translational implications and future research directions*	Page 19, paragraph 3
